# Inoculating science against potential pandemics and information hazards

**DOI:** 10.1371/journal.ppat.1007286

**Published:** 2018-10-04

**Authors:** Kevin M. Esvelt

**Affiliations:** MIT Media Lab, Massachusetts Institute of Technology, Cambridge, Massachusetts, United States of America; University of Pittsburgh, UNITED STATES

## Abstract

The recent de novo assembly of horsepox is an instructive example of an information hazard: published methods enabling poxvirus synthesis led to media coverage spelling out the implications, efficiently disseminating true information that might be used to cause harm. Whether or not the benefits justified the risks, the horsepox saga provides ample reason to upgrade the current system for screening synthesized DNA for hazardous sequences, which does not cover the majority of firms and cannot reliably prevent the assembly of potentially pandemic pathogens. An upgraded system might leverage one-way encryption to confidentially scrutinize virtually all commercial production by a cooperative international network of servers whose integrity can be verified by third parties. Funders could support participating institutions to ease the transition or outright subsidize the market to make clean DNA cheaper, while boycotts by journals, institutions, and funders could ensure compliance and require hardware-level locks on future DNA synthesizers. However, the underlying problem is that security and safety discussions among experts typically follow potentially hazardous events rather than anticipating them. Changing norms and incentives to favor preregistration and advisory peer review of planned experiments could test alternatives to the current closeted research model in select areas of science. Because the fields of synthetic mammalian virology and especially gene drive research involve technologies that could be unilaterally deployed and may self-replicate in the wild, they are compelling candidates for initial trials of early-stage peer review.

Edward Jenner may have used horsepox, not cowpox, to tame the greatest scourge of humanity [1]. The question is whether the de novo synthesis of the horsepox virus [2] will reprise history by inoculating us against a devastating pandemic, this time institutionally, or tragically hasten its arrival.

That the recent assembly of horsepox was physically harmless is not disputed. It is possible that the benefits justified the risks of disclosing dual-use research; this is currently a matter of controversy [3–5]. Regardless, widely publicizing the methodology by which a laboratory can readily assemble a poxvirus is an instructive example of an information hazard: it disseminated true information that could cause harm [6].

The accessibility of large poxviruses was hardly a secret to anyone familiar with synthetic biology, as far larger genomes have been assembled [7,8]. But most people are unlikely to connect synthetic yeast chromosomes to the de novo assembly of truly dangerous viruses, whereas news articles covering horsepox spelled out the principle and possibilities in detail. Rather than arguing over whether horsepox assembly was justified, we should look ahead, for the event highlights systemic flaws in the current research enterprise that impede scientific progress while rendering society increasingly vulnerable to unilateral actions by a few.

## The immediate problem: Improved DNA assembly techniques and awareness of their capabilities have made potential pandemic viruses widely accessible to nonstate actors

Judging by the historical death tolls of natural pandemic viruses, an engineered pandemic could grievously harm civilization [9]. That the horsepox team relied on commercial DNA fragments highlights a key bottleneck limiting potential makers of pandemics: most groups capable of assembling DNA oligonucleotide building blocks cannot synthesize them. Given that viruses generally do not tolerate substantial recoding without severely compromising fitness, adequately screening all synthesized DNA could eliminate the most serious foreseeable hazards of biotech misuse by nonstate actors [4].

**Fig 1:**
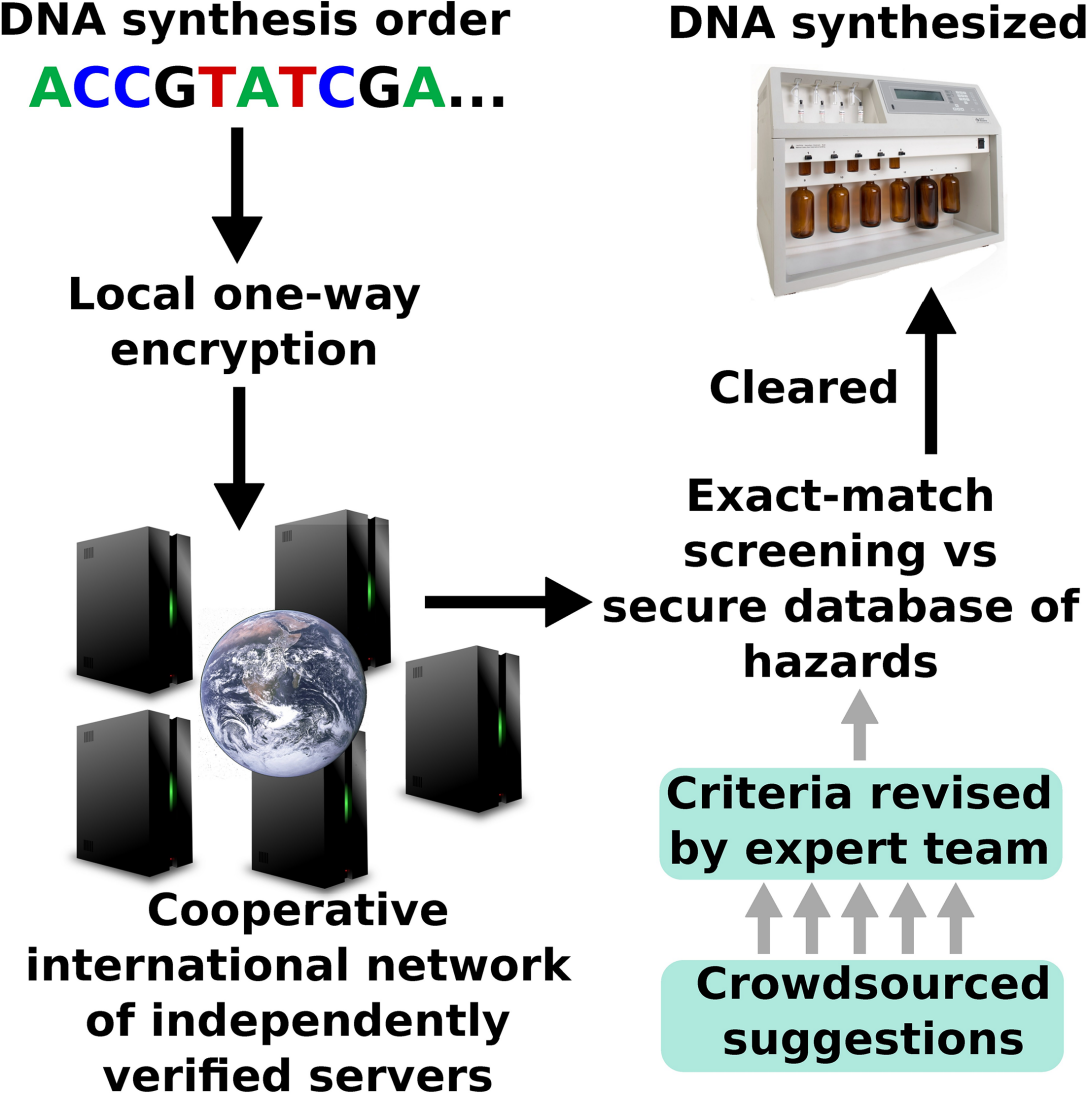
Sketch of a potential improved screening system for DNA synthesis orders. Iterative hashing would enable companies to send out sequences to be screened externally while protecting trade secrets. Order fragments of approximately 40 bp could be screened for exact matches against a hashed database of hazardous sequences by a cooperative international network of servers verifiable by third parties. Orders and database could be kept private using uniquely salted local hashes plus a multiparty ball-and-chain, or possibly homomorphic encryption. Exact sequence comparison and the size of the sequence space relative to order volume could effectively eliminate false positives unless database salting is desired for improved security. Hazardous sequences could be filtered from crowdsourced suggestions by an international team of experts from synthesis companies, universities, and other institutions. Ideally, individual members could add new sequences privately to minimize information hazards.

Today, screening is voluntary and is largely conducted in-house by companies belonging to the International Gene Synthesis Consortium [10], which only covers select agents and leaves approximately 20% of the market unscreened. One possible upgrade might be to employ one-way encryption in order to protect trade secrets while screening exact sequence fragments via an international network of cloud-based servers (Fig 1) [11,12]. The structure and contents of the database should remain private yet be informed by crowdsourced suggestions implemented by an international team of experts familiar with information hazards, each of whom would remain ignorant of sequences added by the others.

**Fig 2:**
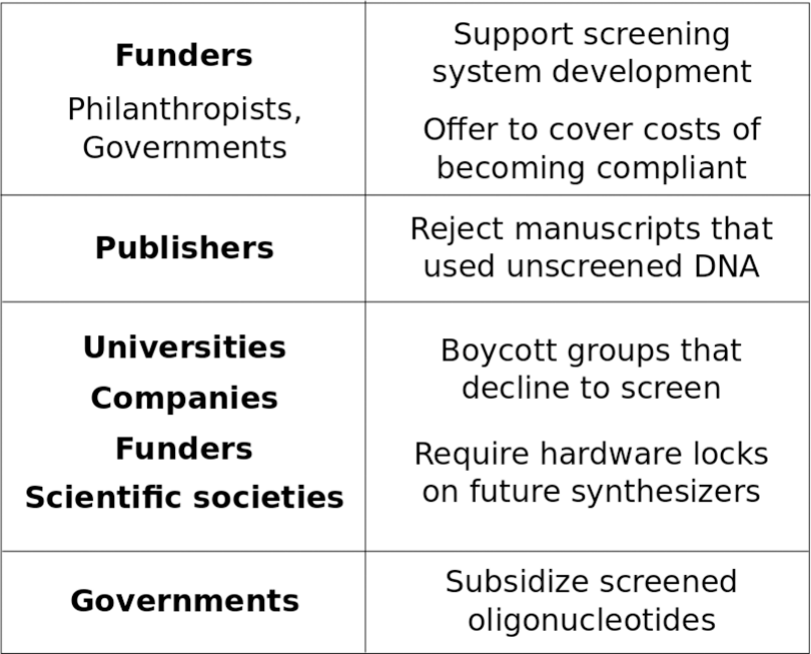
Paths towards the adoption of universal screening of commercial synthetic DNA orders for hazardous sequences. Covering the up-front cost of screening system development and initial adoption would eliminate barriers that might prevent companies from participating. Publishers could incentivize participation by declining to publish submitted manuscripts that rely on unscreened DNA, whereas universities, societies, companies, and funders could boycott groups that decline to adopt screening. Once in place, this system could effectively require hardware-level locks on future hardware permitting distributed synthesis. International governments could subsidize all screened oligonucleotides to impose an effective market-based requirement for approximately 1/15 of the current US annual biodefense budget.

So how do we get from here to there? Ideally, the clear and present danger underlined by the synthesis of horsepox will spur reform (Fig 2). Funders could offer to cover the up-front costs of implementation, whereas journals, professional societies, companies, and universities could help enforce boycotts against providers supplying “dirty” DNA (Fig 2). A 50% subsidy from governments applied to screened oligonucleotides could ensure that “clean” DNA is much cheaper than dirty for approximately US$500 million annually [13] while preserving existing supplier-customer relationships. For comparison, the United States alone spends approximately US$7 billion on biodefense [14]. Once in place, such an incentive structure could then be adapted to cover next-gen methods, even desktop DNA synthesis, by mandating hardware locks requiring cloud-based screening. If those technologies arise first, we will struggle to control misuse.

Implementing a system for universal DNA synthesis screening may require a great deal of effort, but if doing so could take synthetic viral pandemics and many other threats off the table, pitching in may be the most important thing many of us ever do [15]. Unless, that is, we tackle a greater problem.

## The greater problem: Current incentives discourage early peer review, slowing scientific progress and encouraging dissemination of hazardous information concerning increasingly powerful and accessible technologies

Our society appears remarkably inept at reforming poorly adapted institutions until after a disaster [16]. Sadly, that includes the scientific enterprise.

Civilization requires continued scientific progress: we need new advances just to sustain the status quo, let alone to continue the last century’s remarkable gains in health and well-being. But because our current institutions evolved when it was costly to share information, they actively discourage scientists from working together. Sharing ideas and nascent research plans with our peers would let us make informed decisions on candidate projects and whether to collaborate or compete, almost certainly resulting in faster progress. Unfortunately, most scientists quite rationally keep their work to themselves to avoid being “scooped,” necessarily forfeiting the advice of their peers.

Researchers deprived of early advice often make fewer discoveries and are more likely to unwittingly create information hazards. Had other experts known of the planned horsepox research, they could have suggested safer ways to gain the same benefits. For example, the efficacy of horsepox as a vaccine candidate might have been published without mentioning the origin of the virus, whereas research in other subfields might have been catalyzed by assembling a similarly sized virus without an especially deadly relative.

Earlier debates over information hazards involving potentially pandemic pathogens exhibited a similar pattern of safety discussions following—not anticipating—disclosure [17–21]. Whether any of these events were true hazards is not relevant: at some level of technological destructiveness, institutions that discuss safety only after the fact are best described as broken.

Acknowledging these institutional failures should not prevent us from recognizing the heroic work of institutional biosafety committees, which have doubtless prevented countless other errors that consequently never became public. The point is that routinely inviting the advice of expert peers before conducting experiments would likely accelerate scientific progress and minimize information hazards.

That current scientific practices are manifestly suboptimal does not mean we should try to change the incentives overnight. Rather, we might test early peer review models in the most suitable fields: those featuring increasingly accessible dual-use technologies potentially capable of unilaterally affecting large numbers of people.

**Fig 3:**
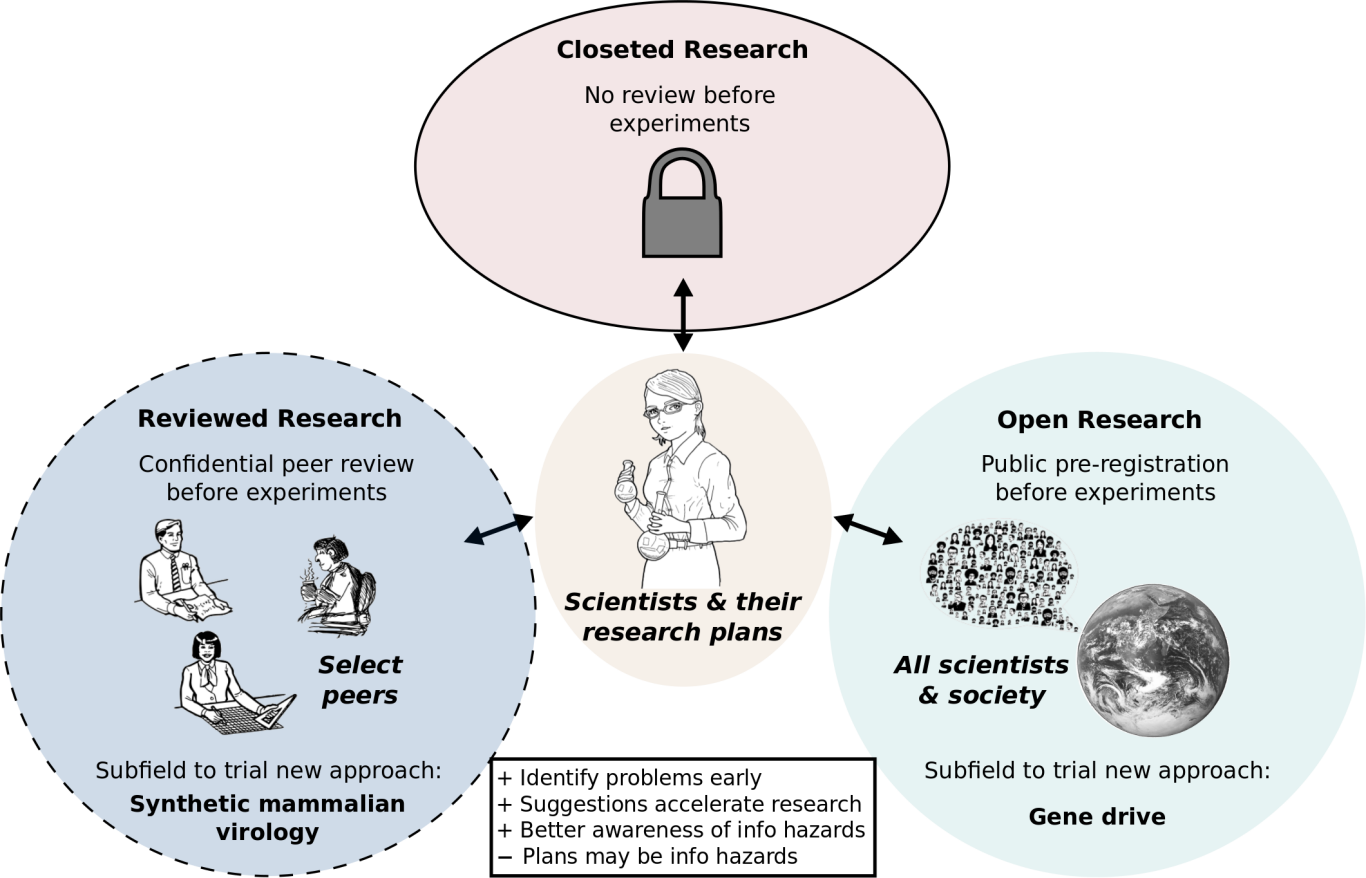
Greater openness could accelerate progress and inoculate science against hazardous mistakes. Current incentives encourage scientists to keep research plans to themselves until publication (top), which prevents others from suggesting improvements. Fields such as gene drive have moral reasons to shift towards a fully open model (right) in which anyone can share advice, but this may not be practical for all fields due to commercial incentives and the risk of disclosing research plans that would themselves be information hazards. An intermediate model (left) might adapt current grant evaluation systems or national boards to ensure that proposed projects are confidentially preregistered and peer-reviewed by experts from diverse fields who lack conflicts of interest, enabling them to suggest ways of mitigating potential hazards in advance of experiments. This approach might be usefully pioneered by the field of synthetic mammalian virology. In both open models, early advice from peers would likely accelerate discovery relative to the current closeted approach.

For example, we detailed CRISPR-based gene drive [22,23], a method of spreading engineered changes through wild populations, only after consulting with experts from a variety of fields [24] who concurred that the technology would be difficult to misuse: it spreads slowly over generations, is easily detected by sequencing, and can be readily overwritten by a subsequent gene drive [25]. They further agreed with our ethical concerns: because gene drives will alter shared ecosystems, research should not be closeted, as people deserve a timely voice in decisions intended to affect them [26]. By publishing and emphasizing the need for early transparency and laboratory safeguards, we hoped to earn public confidence, prevent socially disastrous accidental releases [27], and pioneer a new model of open, preregistered research in a comparatively low-risk field. If successful, this approach could serve as a model for much of science (Fig 3).

But open preregistration may not be suitable for all of science. It is possible to imagine research ideas so hazardous that publicly sharing them in any form would be unwise. In light of horsepox assembly, it may be logical to subject plans involving the de novo assembly of any self-replicating virus capable of infecting mammalian cells to confidential early-stage review. Depending on the nation, existing grant review systems or national boards [28] might be adapted to scrutinize such plans in advance of experiments. Reviewers would suggest improvements and warn of unanticipated problems, potentially accelerating progress and dissuading actions that would generate information hazards.

What can be done to limit the hazards that slip through? At present, we decidedly err on the side of spreading all information. Despite entirely predictable advances in DNA assembly, every human with an internet connection can access the genetic blueprints of viruses that might kill millions. These and worse hazards are conveniently summarized by certain Wikipedia articles, which helpfully cite technical literature relevant to misuse.

Note the deliberate absence of citations in the above paragraph. Citing or linking to already public information hazards may seem nearly harmless, but each instance contributes to a tragedy of the commons in which truly dangerous technical details become readily accessible to everyone. Given that it takes just one well-meaning scientist to irretrievably release a technological information hazard from the metaphorical bottle, it may be wise to begin encouraging norms of caution among authors, peer reviewers, editors, and journalists.

To those for whom these concerns seem excessive: please reconsider. A world in which many individuals can access technologies capable of unilaterally inflicting mass harm is not a world likely to endure. Even if the tree of knowledge does not produce such catastrophic fruits, our ability to conduct research rightfully requires us to earn and maintain public confidence that our work will benefit humanity. Disasters involving new technologies risk not only lives but the opportunity to continue making lifesaving discoveries.

Balancing our civilization’s dependence on continued advances against the hazards posed by powerful and widely accessible technologies may be the defining challenge of our time. Current scientific incentives are ill-advised precisely because they discourage us from receiving early advice from others. Enacting prudent screening strategies, raising awareness of information hazards, and thoughtfully exploring new research models may help accelerate discovery and inoculate science against the development and dissemination of technologies best left alone.
